# Evidence of a new hidden neural network into deep fasciae

**DOI:** 10.1038/s41598-021-92194-z

**Published:** 2021-06-16

**Authors:** Caterina Fede, Lucia Petrelli, Diego Guidolin, Andrea Porzionato, Carmelo Pirri, Chenglei Fan, Raffaele De Caro, Carla Stecco

**Affiliations:** Department of Neurosciences, Institute of Human Anatomy, University of Padua, Via A. Gabelli 65, 35121 Padova, Italy

**Keywords:** Neuroscience, Anatomy

## Abstract

It is recognized that different fasciae have different type of innervation, but actually nothing is known about the specific innervation of the two types of deep fascia, aponeurotic and epymisial fascia. In this work the aponeurotic thoracolumbar fascia and the epymisial gluteal fascia of seven adult C57-BL mice were analysed by Transmission Electron Microscopy and floating immunohistochemistry with the aim to study the organization of nerve fibers, the presence of nerve corpuscles and the amount of autonomic innervation. The antibodies used were Anti-S100, Anti-Tyrosine Hydroxylase and Anti-PGP, specific for the Schwann cells forming myelin, the sympathetic nerve fibers, and the peripheral nerve fibers, respectively. The results showed that the fascial tissue is pervaded by a rhomboid and dense network of nerves. The innervation was statistically significantly lower in the gluteal fascia (2.78 ± 0.6% of positive area, 140.3 ± 31.6/mm^2^ branching points, nerves with 3.2 ± 0.6 mm length and 4.9 ± 0.2 µm thickness) with respect to the thoracolumbar fascia (9.01 ± 0.98% of innervated area, 500.9 ± 43.1 branching points/mm^2^, length of 87.1 ± 1.0 mm, thickness of 5.8 ± 0.2 µm). Both fasciae revealed the same density of autonomic nerve fibers (0.08%). Lastly, corpuscles were not found in thoracolumbar fascia. Based on these results, it is suggested that the two fasciae have different roles in proprioception and pain perception: the free nerve endings inside thoracolumbar fascia may function as proprioceptors, regulating the tensions coming from associated muscles and having a role in nonspecific low back pain, whereas the epymisial fasciae works to coordinate the actions of the various motor units of the underlying muscle.

## Introduction

For many years fascia was considered as an inert tissue, which wraps and gives mechanical support to muscles and other organs. The first demonstrations that fascia is innervated date back to 1957: Stilwell reported some histological findings about sensory nerves in deep fasciae^1^. In 1974, Sakada and co-authors studied the masticatory area but without giving a specific meaning to the presence of mechanoreceptors in the fascia^2^. The first event in which many Authors described a huge presence of sensory nerves in the fasciae with a role in proprioception and nociception was the First International Fascia Congress (2007, Harvard Medical School, Boston)^3^. It is now demonstrated that the different fasciae have different type of innervation: the visceral fascia is rich in autonomic innervation^4^, the superficial fascia shares with the skin mechano- and thermic-receptors, and the deep fascia has a role in proprioception^5^. Furthermore, also different areas show different density and type of innervation. The superficial fascia is the second most highly innervated soft tissue after the skin, with a density of nerve structures of 33.0 ± 2.5/cm^2^ and 64.0 ± 5.2/cm^2^, respectively, and a mean size of 19.1 ± 7.2 μm; the deep fascia has a nerve density of 19 ± 5.0/cm^2^ and presents a thin but huge network of small nerve fibers (mean diameter 15.5 ± 9.4 μm)^6^. Tesarz demonstrated that the different sublayers of thoracolumbar fascia (TLF) have different amounts of nerve fibers, more numerous in the superficial and deep sublayers rather than the middle one^7^.

The two types of deep fascia, aponeurotic and epimysial, have totally different functions and mechanical properties: the aponeurotic fascia envelops various muscles and keep in place and connect them, whereas the epymisial fascia is specific for each muscle and strongly connected with them, defining their form and volume^5^. But despite that, the two type of fasciae are usually considered together and nothing is known about their specific innervation. One of the most studied aponeurotic fasciae is the TLF, being related to non-specific low back pain^8–11^. After chronic inflammation, the density of nociceptive fibers in TLF increased, from 4 to 15%^12^. Schilder^13^ demonstrated that the free nerve endings of TLF are more sensitive to chemical stimulation by injections of hypertonic saline, compared to the underlying muscles and subcutis, maintaining a long lasting hypersensitivity and a longer pain duration. Yahia found corpuscular receptors in human TLF^8^, unlike Mense, who demonstrated in rat TLF the only presence of free nerve endings: they can have both nociceptive and proprioceptive functions, due to a low mechanical threshold^14^. The increase of calcitonin gene related peptides (CGRP) and substance P (SP) positive nociceptive fibers in the inner and outer layers of the inflamed TLF can explain the mechanism of low back pain^10^.

On the contrary, up to now, no studies exist about the innervation of the epimysial fasciae.

The aim of this work was to deeply study the organization of nerve fibers in the deep fasciae: in TLF as aponeurotic fascia, and in gluteal fascia, as epimysial fascia. The use of mouse tissues permitted an exclusive analysis of innervation in a tridimensional way, to better understand the differences in various areas and the presence of autonomic innervation and nerve corpuscles. First of all this work aims to demonstrate that the fasciae are richly innervated tissues, then it allows to evidence for the first time differences in the density of innervation and the presence of corpuscles according to the type of fascia, aponeurotic or epymisial, and the role it plays in proprioception or in coordination of the actions of the motor units.

## Results

**Figure 1:**
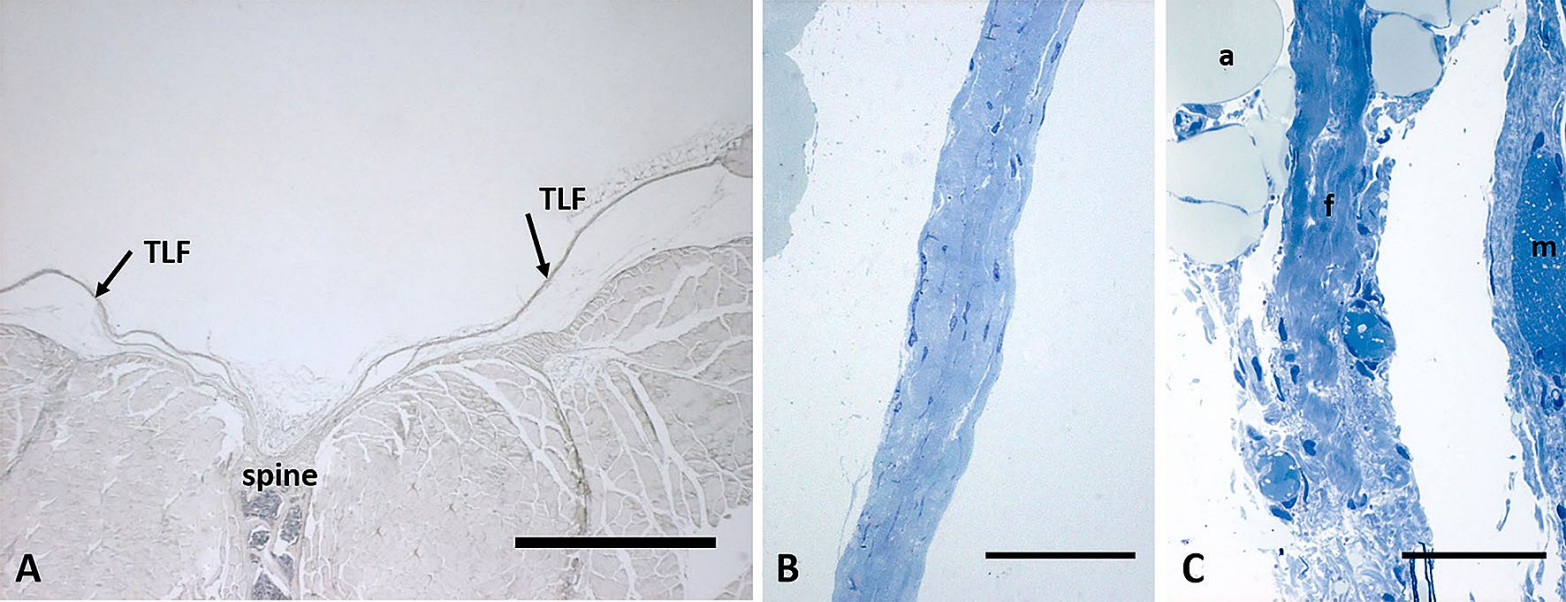
Thickness of thoracolumbar and gluteal fascia: (**A**) 5 µm section of fascia, spine and muscle, stained with ematoxylin and eosin; arrows indicate the thoracolumbar fascia (TLF); scale bar: 1000 µm; (**B**) 0.5 µm semithin section of thoracolumbar fascia stained with Toluidine Blue; scale bar: 50 µm; (**C**) 0.5 µm semithin section of gluteal fascia stained with Toluidine Blue; a: adipocytes; f: gluteal fascia; m: muscle; scale bar: 50 µm.

**Figure 2:**
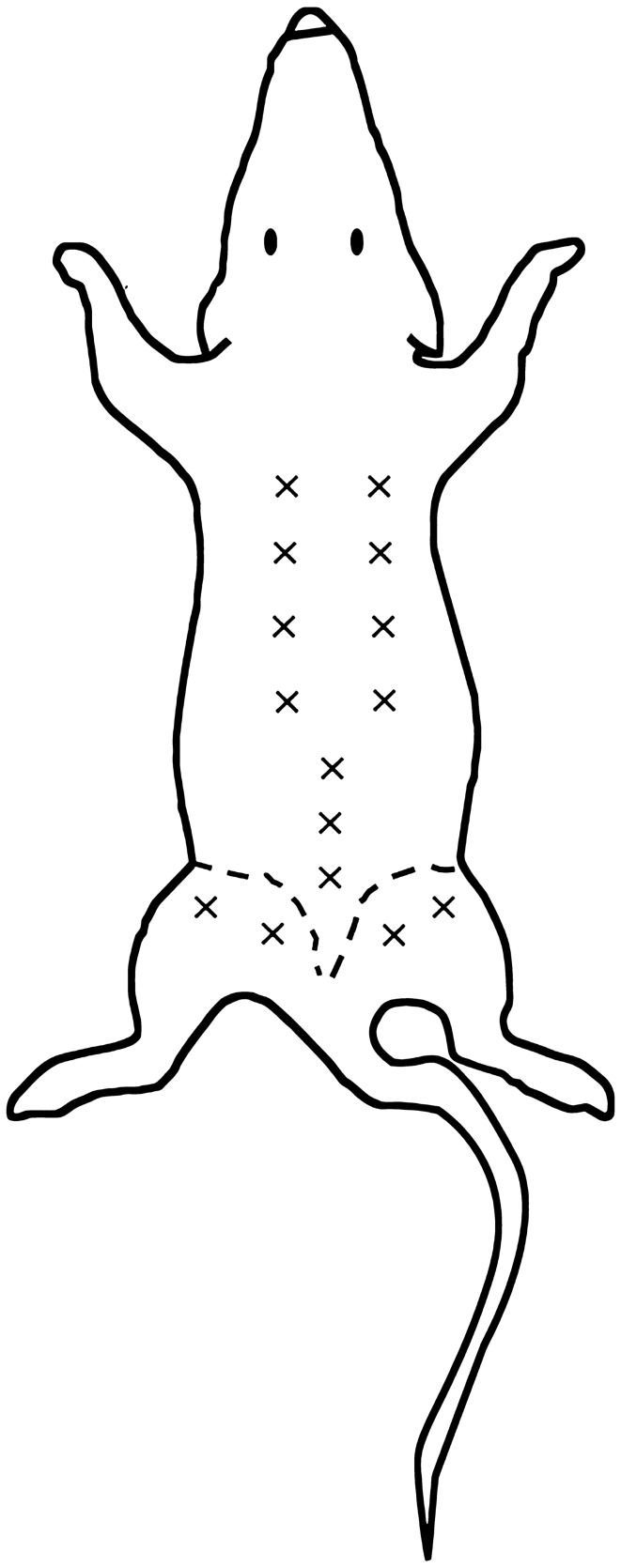
Analysis of density innervation per area and side (right and left): “ × ” indicate each area analyzed after immunohistochemical stains: from thoraco to sacral region of thoracolumbar fascia (both right and left side), and gluteal fascia (right and left side).

**Figure 3:**
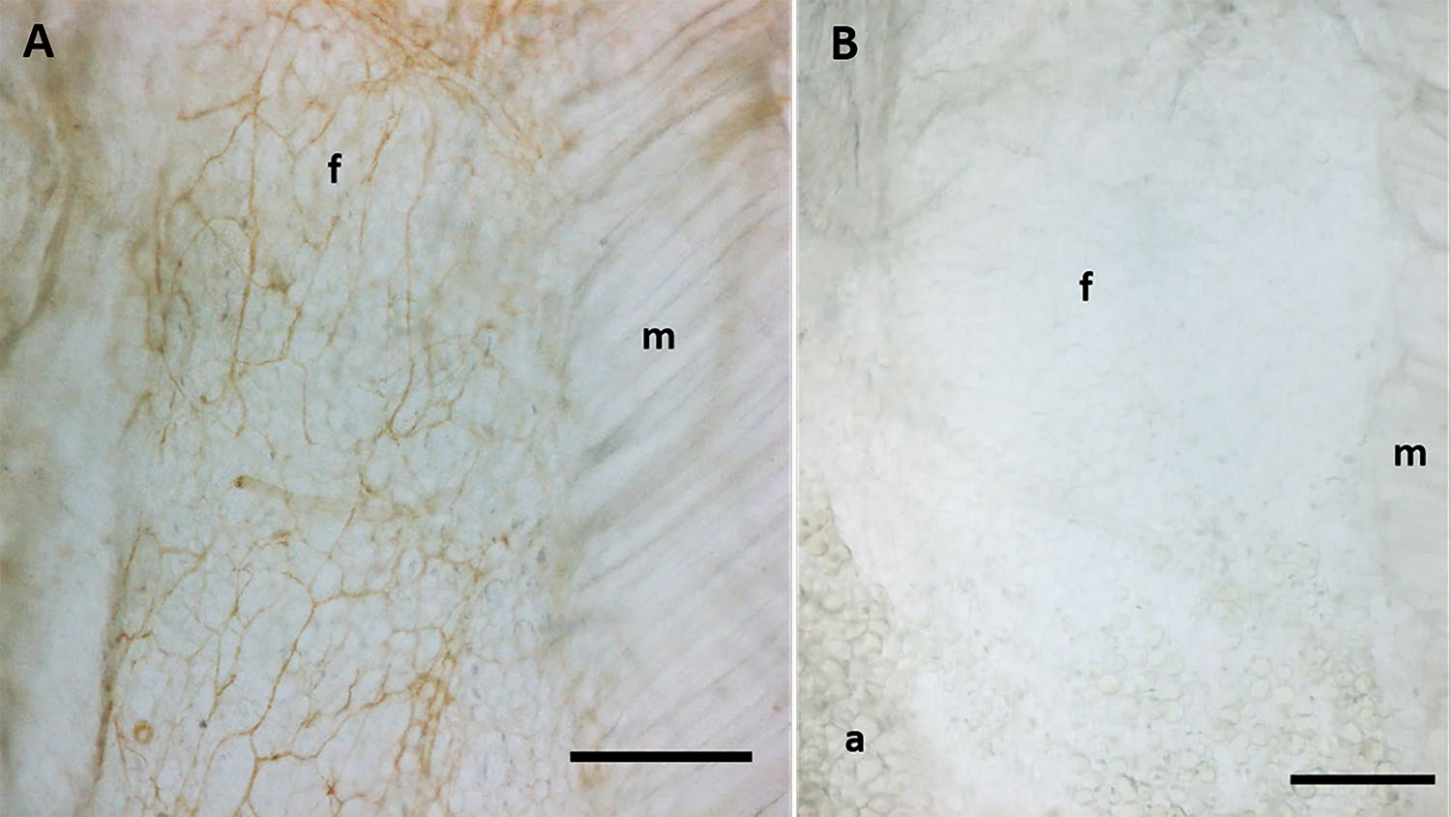
Different density of innervation of fascia and muscle: **A**: S100 immunohistochemistry reaction of thoracolumbar fascia (f) and latissimus dorsi muscle (m). **B**: negative control with the omission of the primary antibody. (a): adypocytes; (f): fascia; (m): muscle. Scale bar: 300 µm.

The mean thickness of the posterior layer of TLF was 34.3 µm ± 12.3 µm, whereas the mean thickness of the gluteal region was 21.3 µm ± 10.4 µm (Fig. 1, analysis performed in seven semithin sections stained with toluidine blue, 5 < areas < 10 for each section, using Image J software). The analysis of density innervation per area or per side (right/left) of TLF and gluteal fascia didn’t highlight any significant difference comparing random areas stained with the same antibody (data not shown): all the areas of TLF, from thoraco to sacral region (identified in Fig. 2), exhibited the same distribution of innervation and the same density; on the other side also the gluteal fascia didn’t show any difference according to the mapped region. Figure 3A shows that the fascial tissue is pervaded entirely by a dense network of nerves individualized by S100 antibody, ending on the border with the muscle, which is not at all equally innervated. The specificity of the staining was demonstrated by the absence of reaction in the negative control (Fig. 3B).

**Figure 4:**
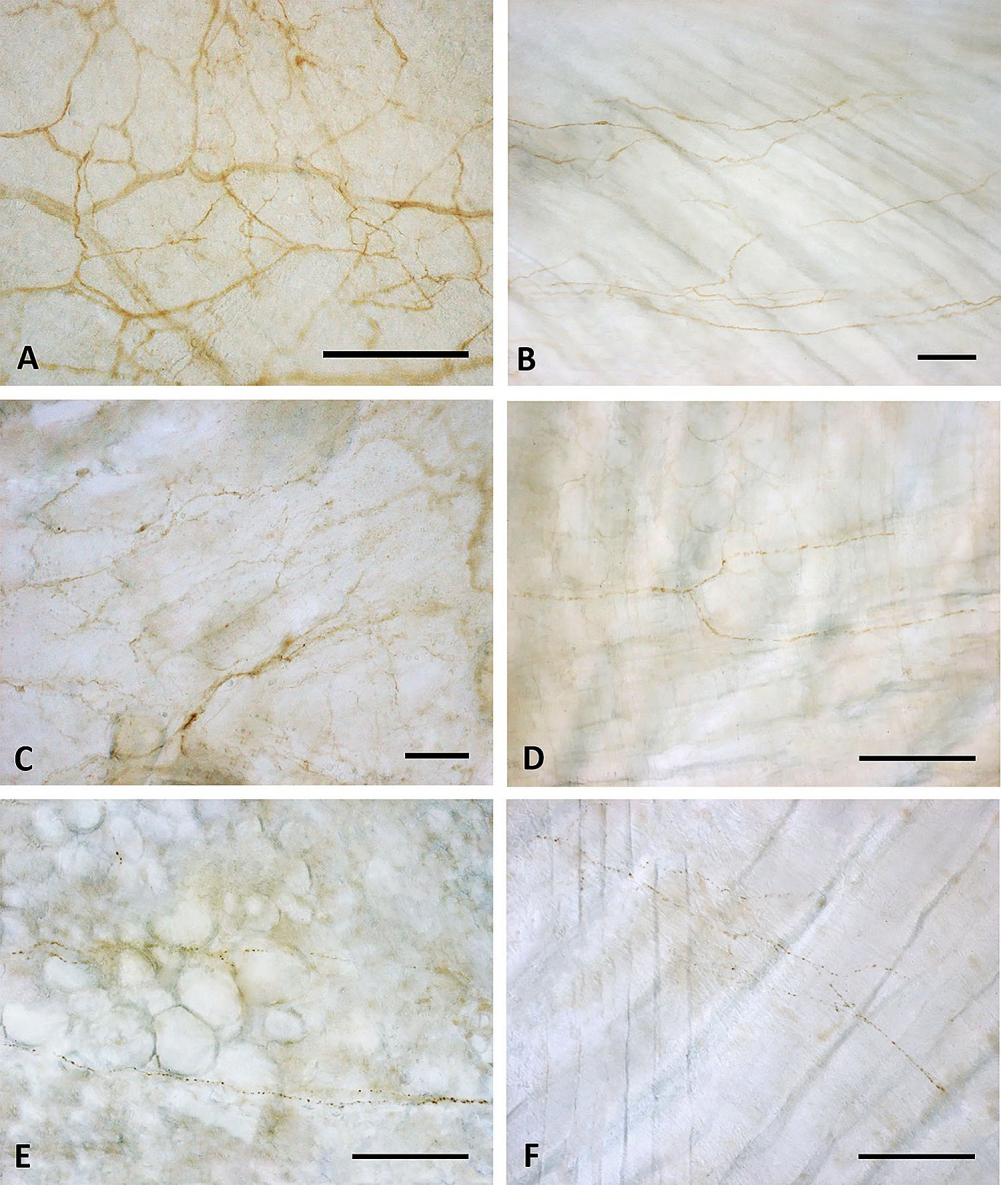
Innervation of thoracolumbar and gluteal fascia: Thoracolumbar fascia (**A**,**C**,**E**) and gluteal fascia (**B**,**D**,**F**) samples stained with S100 (**A**,**B**), PGP 9.5 (**C**,**D**) and Tyrosine Hydroxylase (**E**,**F**) antibodies. Scale bars: 100 µm.

**Figure 5:**
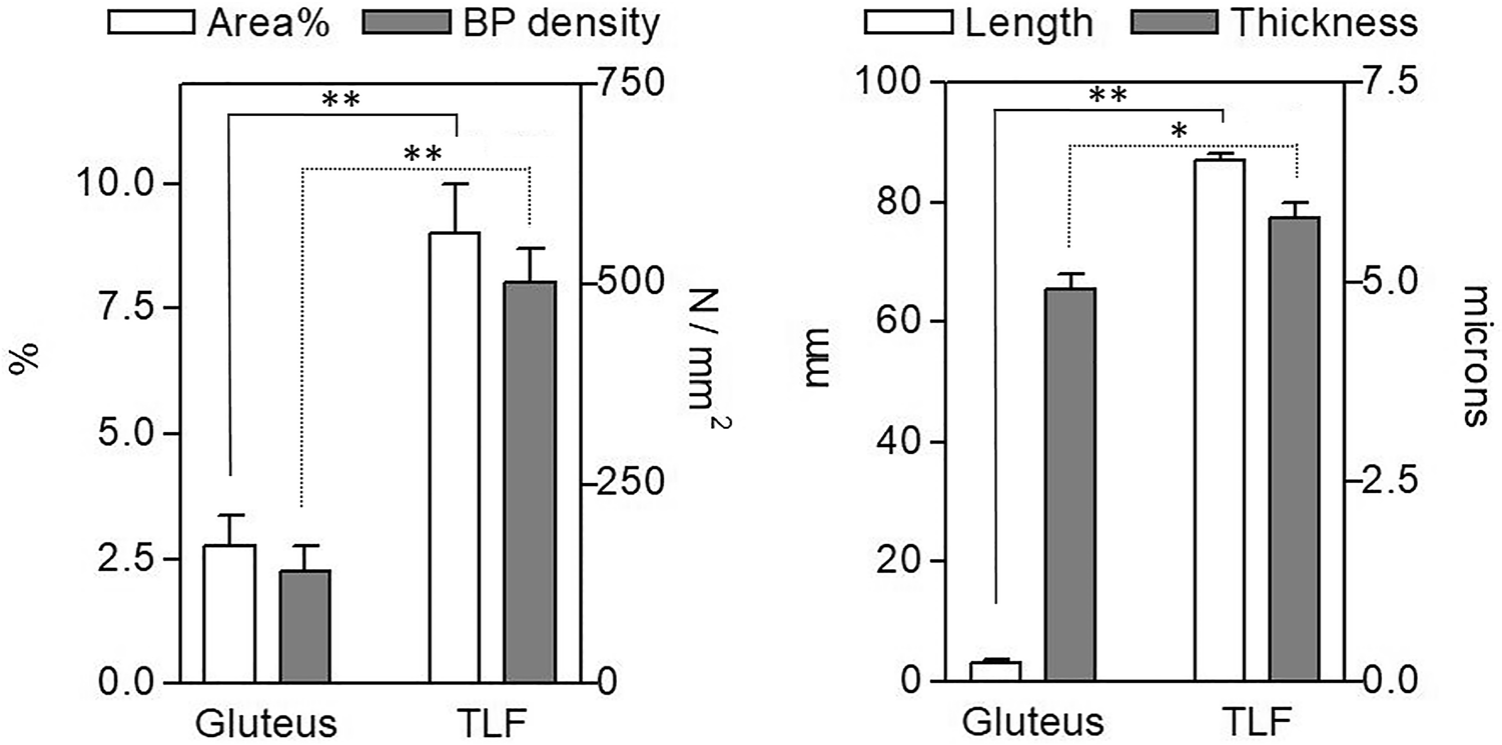
Morphometric analysis of the pattern of S-100 positive fibers in thoracolumbar and gluteal fascia: positive area (%), branching points (BP) density (number/mm^2^), length of nerve structures (mm), thickness of nerve structures (µm) in thoracolumbar fascia (TLF) and gluteal fascia. *p < 0.05; **p < 0.01, *t* test.

Tyrosine Hydroxylase showed less positivity (Fig. 4E–F), with only some positive single nerve filaments. On the contrary, the distribution of PGP 9.5 and S100 reaction were qualitatively similar (Fig. 4C–D,A–B, respectively), with a rhomboid and dense thin network of nerves homogeneously distributed in all the samples area analyzed. The reaction with PGP 9.5 antibody showed, however, thinner positive filaments less contrasted: for this reason the quantitative and morphometric analysis to compare TLF and gluteal fascia were performed on S100-stained images. The results are reported on Figs. 4 and 5. Both by qualitative (Fig. 4B,D,F) and quantitative (Fig. 5) analysis emerged in a clear and evident way that the gluteal fascia was less innervated with respect to the TLF. All the analyzed parameters (percentage of innervated area, density of branching points, length and thickness of the nerve structures) were statistically significantly lower in the epymysial gluteal fascia with respect to the aponeurotic thoracolumbar fascia. In particular, the latter showed a percentage of innervated area equal to 9.01 ± 0.98%, with 500.9 ± 43.1 branching points per mm^2^. Instead in the gluteal fascia the positive area was equal to 2.78 ± 0.6%, with a number of branching points of 140.3 ± 31.6/mm^2^ (p < 0.01, *t*-test gluteal fascia *vs*. TLF). Moreover, the length of the nerves in the gluteal fascia was 3.2 ± 0.6 mm, showing a very statistically significant difference (p < 0.01) with respect to the TLF (mean length 87.1 ± 1.0 mm). In the latter one, also the thickness of the nerve structures was higher (5.8 ± 0.2 µm in TLF *vs* 4.9 ± 0.2 µm in gluteal fascia, p-value < 0.05).

**Figure 6:**
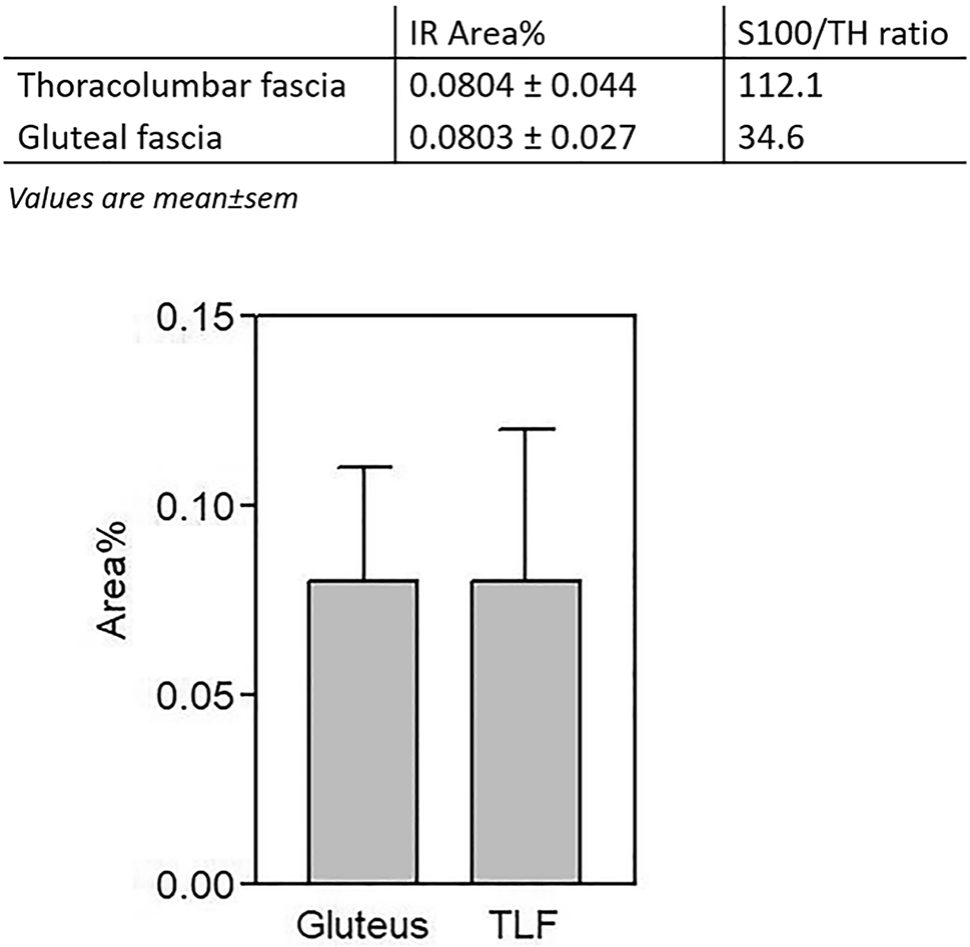
Autonomic innervation in thoracolumbar and gluteal fascia: fraction of area (IR area %) positive to Tyrosine Hydroxylase (TH) and values of S100/TH ratio, in thoracolumbar fascia (TLF) and gluteal fascia, expressed as mean ± standard error mean.

The TH immunohistochemistry showed that in both the fasciae the positive area is around 0.08%, thus leading to a ratio S100/TH positivity of 112.1 in the thoracolumbar fascia and 34.6 in the gluteal fascia (Fig. 6).

**Figure 7:**
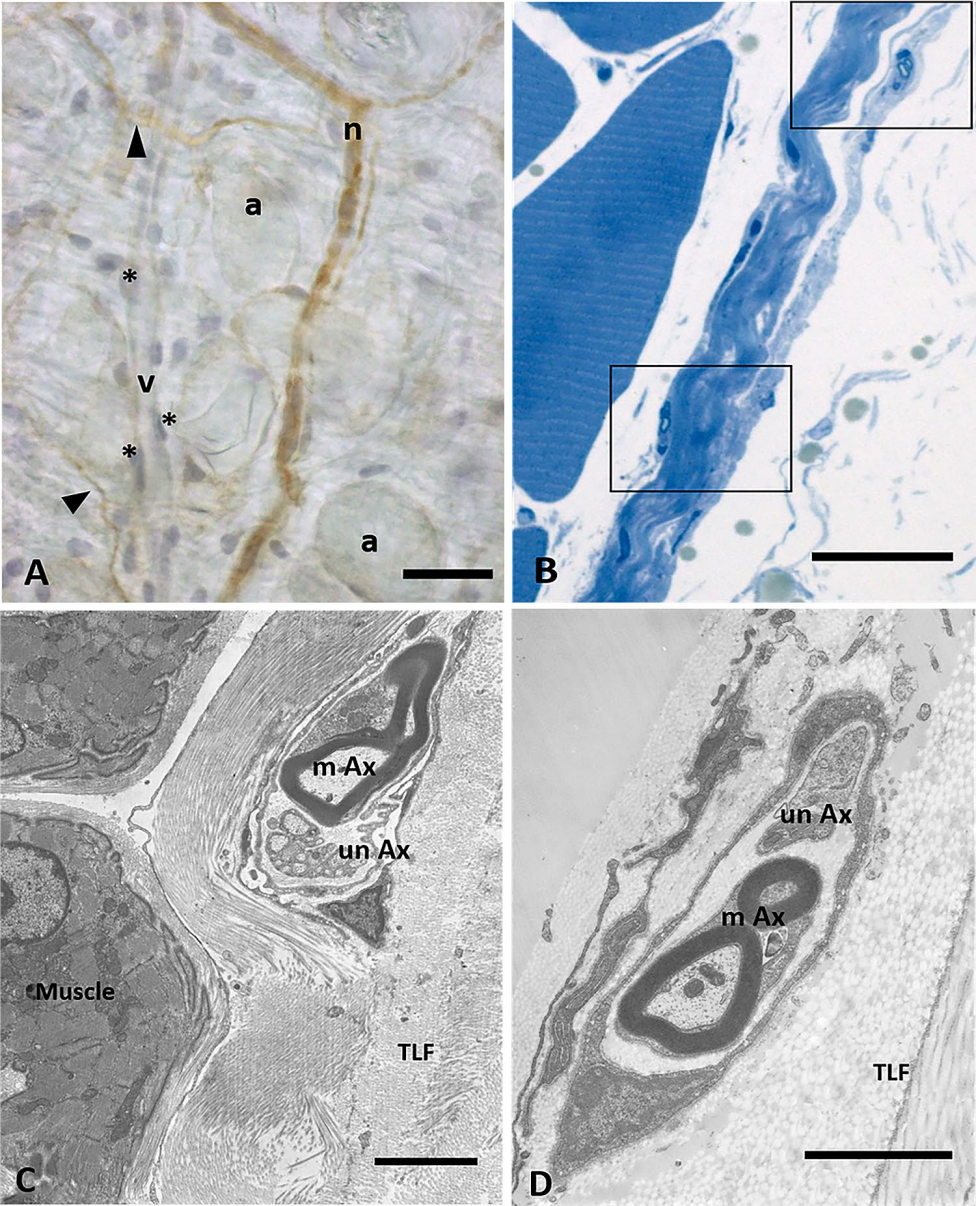
Analysis of nerves inside the fascial tissue: (**A**) Floating thoracolumbar fascia stained with anti-S100 antibody and ematoxylin: the nervous structures are S100 positive (n: small nerve, arrows indicate single nerve fibers), whereas blood vessels are not stained (v: vessel; *: endothelial cells; a: adipocytes). (**B**) Semithin section of thoracolumbar fascia, whose boxes show nerve structures in the midst of collagen bundles of the fascial layers. (**C**) and (**D**): TEM images of a small nerve fiber in the inner layer (**C**) or in the outer layer (**D**) of the TLF, with both myelinc and unmyelinic axons. m: muscle; TLF: thoracolumbar fascia; mAx: myelinic axon; unAx: unmyelinic axon. Scale bars: (**A**) and (**B**) 30 µm; (**C**) 3 µm; (**D**) 2 µm.

A deeper analysis by TEM of the nerves that cross the fascia permitted us to highlight that the majority of the nerve structures (with both myelinic and amyelinic axons) are in the midst of collagen bundles, and not in the muscle (Fig. 7B–C–D) or in the adipose tissue (Fig. 7A).

**Figure 8:**
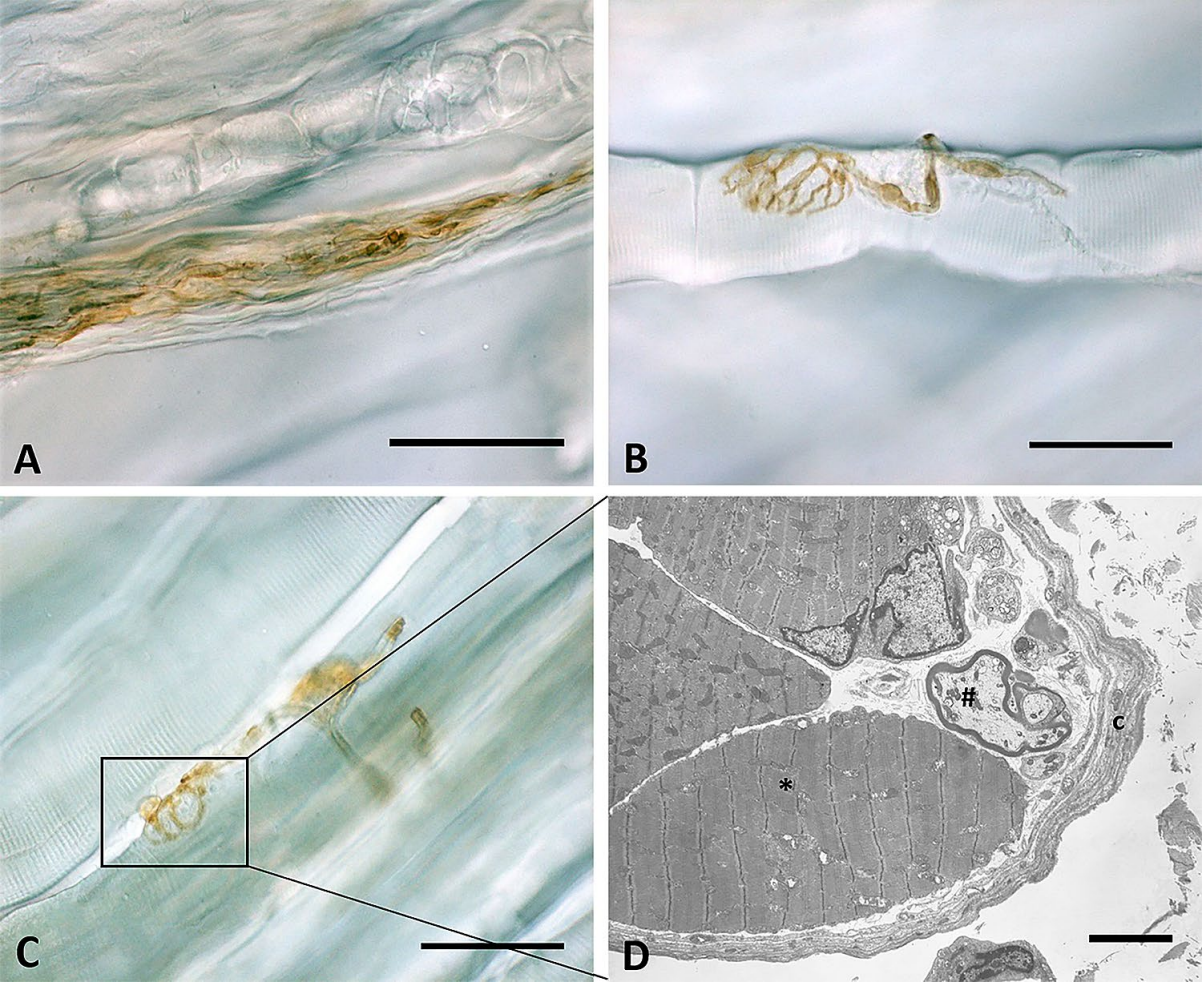
S100-positive corpuscles in gluteal fascia: Golgi tendon organ (**A**), neuromuscular junction (**B**) and muscle spindles (**C**) in gluteal fascia. In (**D**) is shown the TEM analysis of the muscle spindle: a connective tissue capsule (c) wraps a small group of muscle fibers (*) and nerve fibers (#). Scale bars: 50 µm (**A**,**B**,**C**), 5 µm (**D**).

Lastly, the analysis found no presence of any corpuscle in all of the thoracolumbar samples analyzed. In the gluteal fascia, and more specifically in the perimysium and endomysium closely connected to this fascia, Golgi tendon organs (Fig. 8A), neuromuscular junctions (Fig. 8B) and muscle spindles (Fig. 8C–D) have been identified.

## Discussion

This work highlights for the first time the concept that we cannot consider all muscular fasciae as a similar structure. Indeed the TLF and the gluteal fasciae present different densities of innervation with nerve structures of different thicknesses. However, according to our results, both the gluteal and TLF have the same density of autonomic nerve fibers (0.08%). Among all the possible mediators of the autonomic nervous system, we have checked tyrosine hydroxylase that catalyzes the rate limiting step in this synthesis of catecholamines. The positive finding of these fasciae to this enzyme suggests a possible role of the autonomic nervous system in the opening and closing of the vessels inside fasciae, and consequently, it can play a role in the ischemia of fascial tissue. As the amount of sympathetic nerve elements are similar in the two fasciae, we can hypothesize that they are sensitive to ischemia in a similar way. Furthermore, the finding of sympathetic nerve fibers in TLF and gluteal fascia allows to consider that people subjected to a state of chronic stress may have increased levels of pain in those areas, as sympathetic activity is greater under psychological stress^15^. Indeed, it is well recognized that chronic stress can alter the physiological cross-talk between brain and biological systems, leading to compromised functions on the nervous, immune, endocrine, and metabolic systems^16^. This result can help to explain the increased levels of low back pain in patients with chronic stressfull state, a known effect but the mechanisms of which are not yet fully understood^17^.

Although the gluteal and TL fascia present the same density of autonomic innervation, they show a totally different amount of free nerve endings, leading to hypothesize that the major amount is due to the presence of sensitive free nerve endings and suggesting that they can have a different role in proprioception and pain perception. This study also confirms the absence of mechanoreceptors, like Pacini and Ruffini corpuscles, in the thoracolumbar fascia and gluteal fascia, as already demonstrated by Tesarz et al.^7^. It is probable that these receptors are usually localized in the superficial adipose tissue and superficial fascia, with the function of perceiving mechanical stimuli^6^, and where the superficial fascia joints the deep fascia, as in the retinacula surrounding joints, in the palmar and plantar fascia^18–21^, to increase the proprioception of these areas.

The fascial tissue showed a greater and homogeneous distribution of the nerve network with respect to the adjacent muscular tissue (Fig. 3-A). These results are in line with an our previous work about the innervation of the human hip joint, which showed that muscles (vasto-lateral and gluteus medius) were crossed by large nerves bundles (mean number 12 ± 6.1/cm^2^, mean diameter 36.4 ± 13.4 μm), presumably motor nerves, whereas the fasciae were invaded by networks of small nerve fibers (33 ± 2.5/cm^2^, mean size 19.1 ± 7.2 μm in the superficial fascia, and 19.0 ± 5.0/cm^2^, mean diameter 15.5 ± 9.4 μm, in the deep fascia)^22^. These findings lead to the revealing that the pain perception is higher in the fascia with respect to the muscle, highlighting the importance to preserve the fascial structures during surgery and to target to the fascial structures during a manual treatment. It is well know that the TLF could be a source of pain, as demonstrated by Langevin et al.^9^, Tesarz et al.^7^, Schilder et al.^23^, e. g., probably because it can feel the tensions coming from different muscles. Indeed the TLF is not related to a specific muscle, but gives insertions to both the latissimus dorsi, gluteus maximus and external oblique muscles^24^, and also on the inner side it adheres to the serrati posteriors fascia and to the erector spinae aponeurosis^5^. All these muscles stretch the TLF and can create a deformation of the network of the free nerve endings that we have just described. They form a thin and delicate net, strongly connected with the extracellular matrix of the fascial tissue, and consequently are particularly responsive to stretch, shear loading and mechanical stimuli. According to Hoheisel and coauthors^25^ the free nerve endings inside TLF may function as proprioceptors, in the absence of Pacini and Ruffini receptors. Based on this information, we can consider the thoracolumbar fascia as a large proprioceptive element that merges all the tensions coming from its associated muscles. Due to trauma, overuse, poor posture etc., these tensions can become unbalanced, resulting in an anomalous deformation of the free nerve endings, creating deficient motor patterns and eventual pain perceived by the CNS. Bednar et al.^26^ found an alteration in both the histological structure and the degree of innervation of TLF in patients with chronic low back pain. This possibility has surely to be further studied to better explain the possible role of TLF in nonspecific low back pain.

There are minimal studies about the possible role of the gluteal fascia in pain, but the density and type of innervation found in this work suggest that this gluteal fascia is probably less sensitive and consequently plays a minor role as pain generator. The gluteal fascia is totally adherent to the underlying muscle and is connected with some muscle spindles and Golgi corpuscles. These nerve corpuscles are specialized to feel the state of contraction of the various muscular bundles and to regulate them. It is well known that their capsules are totally in continuity with the perimysium and endomysium of the muscle^27^, and consequently any alteration of the intramuscular connective tissue can alter the sensitivity of these receptors, as demonstrated for dystrophy^28^. We also know that the intrafusal spindle muscular fibers can stretch the surrounding connective tissue, and consequently through fascial continuity among endomysium, perimysium and epimysium affect the tension of the gluteal fascia. Therefore, we can hypothesize that the nerve network in the gluteal fascia can unify all the tensions coming from the muscle spindles of the gluteus maximus muscle and transmit by way of the Central Nervous System a decoded input about the state of contraction of the gluteus maximus. So, we can suggest that the epymisial fasciae works more to coordinate the actions of the various motor units present in the underlying muscle, rather than just regulating the tensions originating from many directions as the TLF does.

## Methods

### Samples collection

**Figure 9:**
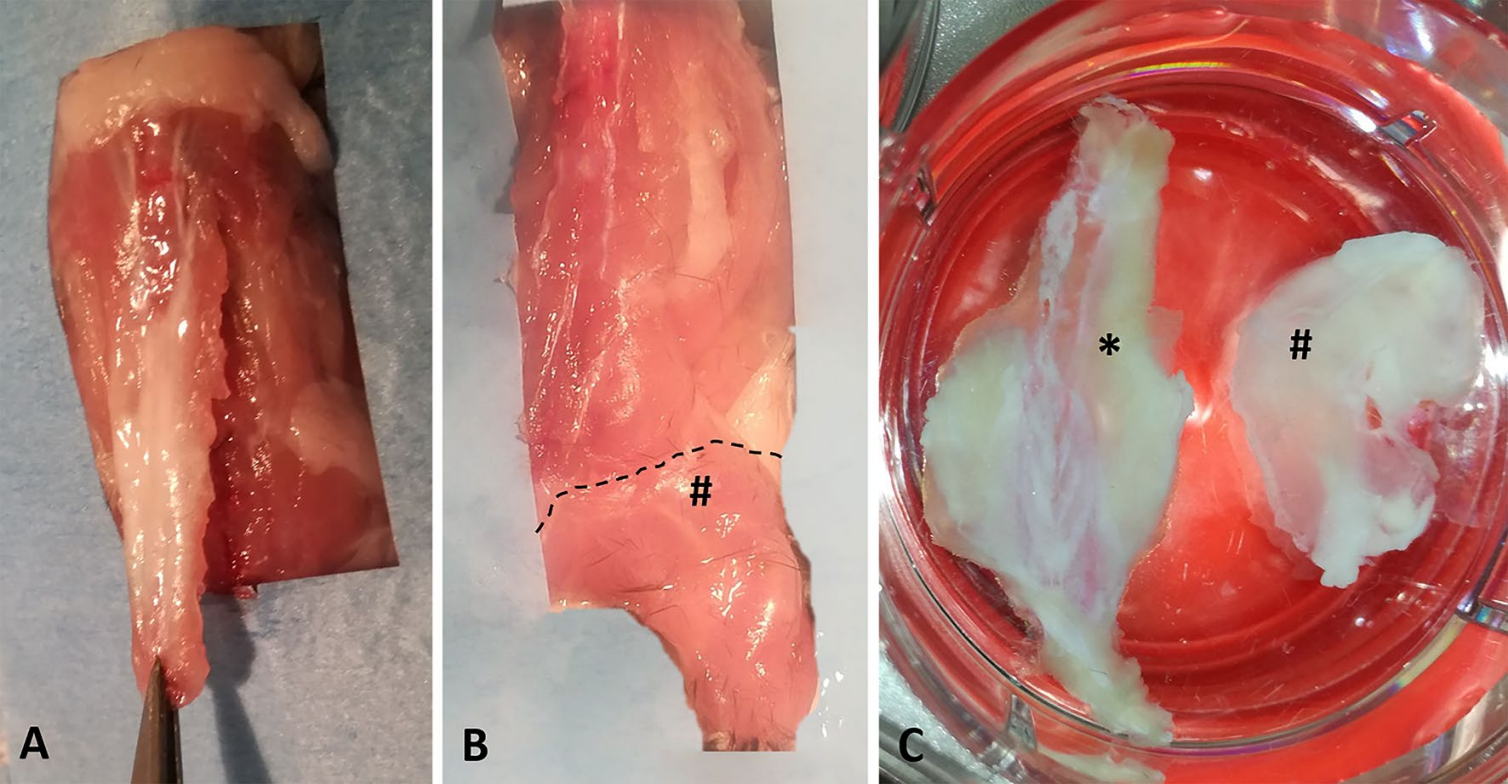
Samples collection and macroscopic images of thoracolumbar fascia and gluteal fascia: (**A**) collection of thoracolumbar fascia; (**B**) collection of gluteal fascia (#); (**C**) floating samples of thoracolumbar fascia (*) and gluteal fascia (#).

The experiments were performed on seven adult C57-BL mice (all males, mean age 8 weeks). All animal procedures were approved by the ethical committee of the University of Padova, in agreement with the guidelines of the Italian Department of Health, and in compliance with the ARRIVE (Animal Research: Reporting of In Vivo Experiments) guidelines. The specimens were collected from Thoracolumbar Fascia (TLF), as an example of the aponeurotic fascia, and gluteal fascia (GF), as an example of epimysial fascia. The posterior layer of the TLF, from the sacral to the thoracic region was separated from the spine (Fig. 9A) and maintained as an unique specimen with its orientation during all the steps. The gluteal fascia layer was removed from the sacral column to the gluteal region (Fig. 9B).

The fascia collected from one mice was fixed for 24 h in 10% formalin solution and for two weeks in 10% EDTA, then embedded in paraffin and cut in four-μm-thick sections for classical histological analysis and ematoxylin stains (Fig. 1-A). The fascia tissues from the others six mices were divided in three parts: one small piece of tissue (2 × 5 mm) was collected, subdivided in 15 small fragments and processed for semithin sections analysis and TEM analysis (see Transmission Electron Microscopy paragraph), and two were processed for Immunohistochemistry floating reaction (Fig. 9C), using randomly one of these for S100 staining (total of six samples), and one for TH or PGP stainings (three samples for each antibody), following the protocol described in the paragraph below.

### Immunohistochemical method

The floating fascia samples of thoracolumbar and gluteal region were fixed in formalin 10% in Phosphate Buffer Saline (PBS). After repeated washings in PBS, endogen peroxidases were blocked with 1% H_2_O_2_ in PBS for 20 min at room temperature. The floating tissues were then pre-incubated with a blocking buffer (BSA 0.1% in PBS) for 60 min at room temperature and then incubated in rabbit polyclonal Anti S100 (Dako, dilution 1:4000), Rabbit Anti Tyrosine Hydroxylase (TH, GeneTex, dilution diluited 1:700), Rabbit Anti PGP (GeneTex, dilution 1:500) in the same pre-incubation buffer and maintained overnight at 4 °C. We selected these antibodies because S100 is specific for Schwann cells forming myelin, TH stains the sympathetic nerve fibres, the anti-PGP is widely used as a marker for all peripheral nerve fibers. After repeated PBS washing, the floating thoracolumbar and gluteal fasciae were incubated for 1 h in goat anti rabbit HRP (Jackson ImmunoResearch—Laboratories, Inc., Cambridge -UK) diluted 1:300 in the same pre-incubation buffer and washed in PBS. Negative controls underwent the same protocol steps, with the omission of the primary antibodies. The reaction was then developed with 3,3′-diaminobenzidine (Liquid DAB + substrate Chromogen System kit Dako Corp, Carpinteria, CA, USA) and stopped with distilled water.

### Image acquisition

The images were acquired by using Leica DMR microscope (Leica Microsystems, Wetzlar, Germany).

For the image processing at least 10 pictures for each sample were acquired at 40X enlargement, to analyze the pattern of nerve fibers. Each sample was also entirely analyzed under light microscope to visualize any corpuscles.

### Image processing and analysis

**Figure 10:**
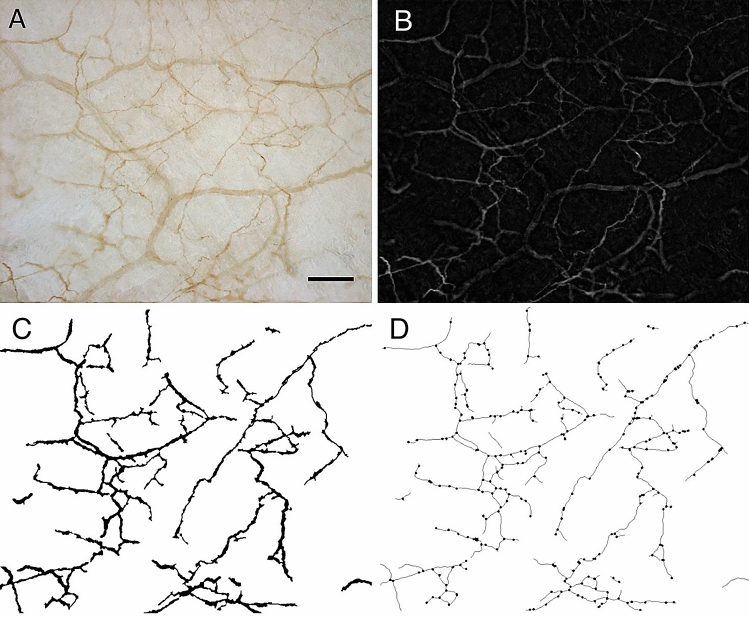
Images processing and morphometric analysis of the pattern of nerve fibers: (**A**) original picture of one S100 reaction; (**B**) top-hat filter applied in picture A to enhance the contrast; (**C**) binary image of nerve network; (**D**) final binary skeleton derived from picture C and identification of the branching points. Scale bar: 100 µm.

To morphometrically characterize the pattern of nerve fibers, the images of S100-stained samples of thoracolumbar and gluteal fascia underwent steps of image processing and analysis performed with ImageJ software as illustrated in Fig. 10.

Briefly, after shading correction, removal of background signals and manual adjustment of each image to eliminate non-nervous structures, a top-hat filter^29^ was applied (Fig. 10B) to enhance the contrast between the pattern of nerve fibers and the background. An adaptive discrimination procedure^30^ was then applied to select nerve profiles. This method operated with a local threshold: the mean grey value of a neighboring region was calculated for every pixel (by a 15 × 15 pixel low-pass filter) and this value plus an offset threshold constant defined the local threshold for that pixel. After interactive editing of the remaining artifacts, a binary image of the nerve network was then obtained (see Fig. 10C). By using binary thinning procedures^31^ the binary skeleton of this image was finally derived (Fig. 10D) and the branching points identified^32^. Nerve density was estimated from the binary image by evaluating the area fraction covered by the fibers, while from the binary skeleton image the total length of the nerve network and the density of branching points were estimated. From these primary morphometric parameters, the mean thickness of the nerve fibers was also derived as the ratio between the area of the network and its length.

Furthermore, nerve density was estimated also from images of TH-stained samples to estimate the fraction of area (%) positive to Tyrosine Hydoxylase and to calculate the S100/TH ratio.

### Statistical analysis

Student’s *t* test was used to verify significant differences when comparing data of area of positivity to S100 (%), branching points density (number/mm^2^), length (mm) and thickness (µm) of the nerve structures, and relative percentage of positivity of tyrosine hydroxylase of the thoracolumbar fascia and the gluteal fascia.

### Transmission electron microscopy (TEM)

The specimens of thoracolumbar and gluteal fasciae were fixed in 2.5% glutaraldehyde (Serva Electrophoresis, Heidelberg, Germany) in 0.1 M phosphate buffer, pH 7.3, and post-fixed in 1% osmium tetroxide (Agar Scientific Elektron Technology, Stansted, UK) in 0.1 M phosphate buffer, dehydrated in a graded alcohol series and then embedded in Epoxy Embedding Medium Kit (45349, Sigma-Aldrich, St. Gallen, Switzerland). Semithin (0.5 µm) and ultrathin (60 nm) sections were cut with the ultramicrotome RMC-PTX PowerTome (Boeckeler Instruments, Arizona –USA). Semithin sections were stained with 1% Toluidine blue solution. Ultrathin sections were collected on 300-mesh copper grids, counterstained with 1% uranyl acetate and then with Sato’s lead. Specimens were observed by a Hitachi H-300 Transmission Electron Microscope (Japan).

## Data Availability

All data generated or analysed during this study are included in this published article.
